# Investigation of the Therapeutic Potential of Boric Acid in Enteritis‐Induced Quails

**DOI:** 10.1002/vms3.70481

**Published:** 2025-07-18

**Authors:** Sultan Aslan, Ulku G. Simsek, Hatice Eroksuz, Burcu Karagulle, Mehmet Eroglu, Gokhan K. Incili, Canan A. Incili

**Affiliations:** ^1^ Department of Animal Science Faculty of Veterinary Medicine Dicle University Diyarbakir Türkiye; ^2^ Department of Animal Science Faculty of Veterinary Medicine Firat University Elazig Türkiye; ^3^ Department of Pathology Faculty of Veterinary Medicine Firat University Elazig Türkiye; ^4^ Department of Microbiology Faculty of Veterinary Medicine Firat University Elazig Türkiye; ^5^ Department of Animal Science Faculty of Veterinary Medicine Siirt University Siirt Türkiye; ^6^ Department of Food Hygiene and Technology Faculty of Veterinary Medicine Firat University Elazig Türkiye

**Keywords:** antibiotic, boric acid, enteritis, intestine, health

## Abstract

This study investigated the effects of boric acid added to feed and water on intestinal histopathology, immune parameters, and intestinal bacterial flora in experimentally enteritis‐induced Japanese quails. Eighty‐four quails were used in the study, which were divided into seven groups. The following groups were used in the study: control (G1), infected (G2), infected + antibiotic (G3), infected + boric acid added to feed (100 mg/kg) (G4), infected + boric acid added to feed (300 mg/kg) (G5), infected + boric acid added into water (100 mg/L) (G6) and infected + boric acid added into water (300 mg/L) (G7). Data were analysed using one‐way ANOVA, and differences between groups were determined by Tukey's honestly significant difference test. The highest levels of white blood cells were found in the infection group, whereas the lowest levels were found in the control group (*p* < 0.05). Boric acid taken with water decreased the number of *Enterobacteriaceae, Escherchia coli*, and total mesophilic aerobic bacteria. However, these differences were not significant compared with the infection group (*p* > 0.05). Compared to the Infected group (G2), the most notable reductions in coliform counts were observed in the boric acid water 300 mg/L group (G7), which decreased from 3.29 to 2.90 log CFU/g (*p* < 0.01), and in the 100 mg/L group (G6), which showed a decrease to 2.93 log CFU/g (*p* < 0.01), In contrast, boric acid given in feed (G4 and G5) had no significant effect on any bacterial count compared to the infected group *p* > 0.05). Consequently, Effective results were observed when boric acid was added to drinking water, even with short‐term (7 days) use. Boron derivatives could provide an effective alternative treatment option, especially in combating the growing antibiotic resistance.

## Graphical abstract

This study examined the effects of boric acid in feed and water on intestinal health and bacterial flora in enteritis‐induced Japanese quails. Boric acid added to drinking water significantly reduced coliform counts, especially at 300 mg/L (*p* < 0.01), while feed supplementation showed no effect. These findings suggest boric acid in water as a promising alternative to antibiotics for controlling intestinal infections and antibiotic resistance.

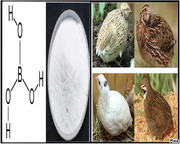

## 1 Introduction

Poultry farming is a key component of animal husbandry, offering a fast and economical means of producing high‐quality animal products. Eggs, in particular, have long been recognized as one of the most affordable and nutritious sources of animal protein. Notably, poultry has become one of the fastest‐growing sources of meat globally, accounting for nearly a quarter of total meat production by the year 2000. This growth has been driven by advancements in genetic selection, improved nutrition, and enhanced health management practices, including the widespread use of antibiotics to treat bacterial infections in intensive farming systems. Historically, antibiotics have also been employed as growth promoters to meet the increasing global demand for food. However, growing public concern over the potential health risks associated with antibiotic use—particularly the emergence of antibiotic‐resistant microorganisms—has led to regulatory restrictions and outright bans in many countries. As a result, attention has shifted toward safer and more sustainable alternatives. A range of substitutes is currently being investigated and applied, including antibodies, bacteriophages, vaccines, organic acids, enzymes, plant‐derived compounds, essential oils, prebiotics, and trace elements such as chromium. Among these, boron stands out due to its promising biological properties (Apata 2009; Kheiri, and Toghyani 2009; Romero‐Aguilar et al. 2019; Eroglu et al. 2024).

Boron (B) is a semi‐metalloid trace element found in nature bound to sodium, magnesium, calcium, and their oxides. These minerals are called boron minerals or boron salts. The simplest boron compounds are boron oxide and boric acid. It has been revealed that boron plays key roles in lipid metabolism, mineral metabolism, energy metabolism, the immune system, the endocrine system, and the nervous system (Söğüt and Acar 2020; Sarı and Soysal 2021). Thanks to its properties, boric acid has demonstrated several beneficial effects when used as a dietary supplement in poultry. These include improved egg weight, eggshell thickness, feed conversion efficiency, and body weight gain (Ayasan et al. 2011; Pradhan et al. 2020). Another important feature of boron is its antimicrobial properties (Celebi et al. 2024). It has been demonstrated that different derivatives of boron have antimicrobial activity on Gram‐positive, Gram‐negative bacteria and yeasts under in vitro conditions, especially on many species such as *Staphylococcus*, *Streptococcus, Candida*, *Escherichia*, *Acinetobacter*, and *Pseudomonas* (Kıvanç et al. 2018; Szałaj et al. 2018; Ateş et al., 2024). Furthermore, these antimicrobial properties may enhance hatching outcomes by reducing the harmful microorganisms on the eggs (Eroglu et al. 2025). Apart from its well‐known antimicrobial properties, boron holds great promise in the development of next‐generation antibiotics. Along with its proven efficacy against resistant pathogens such as *Escherchia coli* (*E. coli*) and *P. aeruginosa*, its unique ability to substitute for conventional elements like calcium in drug design offers new opportunities for structural and functional innovations in antimicrobial therapies (Hernandez et al. 2013; Chiou et al. 2024).

The gastrointestinal tract's microbial load (microbiota) plays a crucial role in health and well‐being. Various factors, including nutritional, stress, and infectious factors, affect the microbiota. Changing gut microbiota in favour of harmful microorganisms can result in enteritis and health problems. A delicate balance exists between immune cells and beneficial bacteria to prevent harmful bacteria in the gut from causing potential inflammation. White blood cells (neutrophils, lymphocytes, eosinophils, monocytes, basophils, etc.), which are important immune system elements, protect the organism by fighting against inflammatory agents in the organism. In inflammation, stimulation occurs within minutes, and the response peaks in 24–48 h (Radi 2004; Cam and Guven 2019). Romero‐Aguilar et al. (2019) reported that boron‐containing compounds are effective in inflammation caused by infectious and non‐infectious agents and provide a therapeutic effect on pathological changes caused by these agents. Moreover, studies have shown that boron bolsters the antioxidant defence system by increasing the production of catalase, superoxide dismutase, and glutathione, which has an immune‐stimulant effect. Besides, it has been determined that boron has an anti‐inflammatory effect by decreasing the mRNA expression levels of  Bax proteins and suppressing TNF‐α production induced by lipopolysaccharides. Furthermore, it has also been found to have an epithelizing effect by maintaining mucosal integrity in cases of enteritis (Kuru &Yarat 2017; Nielsen 2017).

The present study investigated the therapeutic effects of boric acid, administered in different doses through feed and water as an alternative to antibiotics, on intestinal pathological changes, immune parameters, and intestinal bacterial flora in Japanese quails with experimentally induced enteritis.

## 2 Materials and Methods

### 2.1 Experiment Design

The experiment was conducted on 84 Japanese quails (Coturnix japonica), where enteric infection was successfully induced. The sample size was determined based on similar experimental infection studies conducted in Japanese quails (Mazlan et al. 2017; Bertran et al. 2013). All procedures were carried out in environmentally isolated cages to prevent external contamination. An automated lighting system was implemented, providing 16 h of light and 8 h of darkness daily. Feed and water were supplied ad libitum. The composition and nutritional content of the basal diet are presented in Table 1.

**TABLE 1:** Composition and nutritional values of basal diet^c^.

Feed ingredients	%	Nutrients	%
Sweetcorn	51.40	Dry matter	90.40
Wheat bran	9.00	Crude protein	18.00
Soybean meal (% 44 HP)	22.00	Crude fiber	4.40
Corn germ meal	2.00	Crude oil	5.35
Sunflower meal (% 45 HP)	4.30	Crude ash	10.19
Vegetable oil	3.50	Calcium	2.50
Calcium phosphate	0.88	Phosphorus	0.35
Calcium carbonate	4.50	Sodium	0.18
Limestone	1.43	Lysine	1.00
l‐lysine hydrochloride	0.16	Methionine + cystine	0.59
l‐threonine	0.12	Threonine	0.76
Sodium bicarbonate	0.16	Tryptophan	0.25
Salt	0.20	ME, kcal/kg^b^	2800
Vitamin‐mineral mix^a^	0.35		

^a^ Vitamin‐mineral mix (per 1 kg): vitamin A 15,500 IU; vitamin D3 3,500 IU, manganese 120 mg; zinc 100 mg; copper 16 mg; iron 40 mg; iodine 1.25 mg; selenium 0.30 mg; It contains 200 mg of cobalt.

^b^ Determined by calculation.

^c^ The properties of boron used in the research are as follows: Purity >99, borontrioxide (B_2_O_3_) >56.0; sulfate (SO_4_) <0.02; chlorite (Cl) <0.001; iron (Fe) <0.0007

The following groups were used in the study: Control (G1), infected (G2), infected + antibiotic (G3), infected + boric acid added to feed (100 mg/kg) (G4), infected + boric acid added to feed (300 mg/kg) (G5), infected + boric acid added into water (100 mg/L) (G6), and infected + boric acid added into water (300 mg/L) (G7).

The experimental infection was induced using the Escherichia coli O157:H7 ATCC 43895 reference strain, which is associated with hemorrhagic colitis and hemolytic‐uremic syndrome (Reinders et al. 2001; Peroutka‐Bigus et al. 2024). The presence of the strain was confirmed by conventional methods and polymerase chain reaction (PCR) using species‐specific primers, as described by Sheng et al. (2011). A preliminary study was conducted to determine the infection dose, testing 1.5 × 10^7^, 1.5 × 10^8^, and 1.5 × 10^9^ CFU/mL, and based on these results, a dose of 1.5 × 10^8^ CFU/mL was selected to induce infection. To prepare this dose, 100 µL of the bacterial stock, stored at − 20°C, was aseptically inoculated onto blood agar plates supplemented with 5% defibrinated sheep blood to facilitate bacterial growth. Plates were incubated aerobically at 37°C for 24 h to obtain fresh, viable colonies. Subsequently, bacterial colonies were harvested and suspended in sterile 0.9% physiological saline. The suspension was adjusted to an optical density equivalent to 0.5 McFarland standard, corresponding to approximately 1.5 × 10^8^ CFU/mL, measured spectrophotometrically at 600 nm. Bacterial concentration was verified by serial dilution and plating on MacConkey agar to ensure accuracy. Each quail was orally inoculated with 2 mL of the bacterial suspension via sterile gavage needles to simulate natural infection.

Post‐inoculation, quails were monitored for clinical signs including lethargy, diarrhoea, and ruffled feathers. The experimental protocol commenced following histopathological confirmation of enteric infection in intestinal tissue specimens, characterized by definitive enteritis lesions, using animals excluded from the main experimental groups. After infection induction, quails underwent continuous clinical monitoring for signs such as piloerection, lethargy, depression, diarrhoea, and associated pathological changes. Upon confirmation of the infection, boric acid supplements were administered via feed and water, and a commercial preparation containing colistin, an antibiotic effective against *E. coli*, was administered to the G3 group via drinking water at a dosage of 115 mg/kg body weight, by the manufacturer's instructions. All quails were euthanized one week after and tissue samples from the intestinal mucosa were collected and examined histopathologically to evaluate enteritis‐associated changes.

### 2.2 Histopathological Analysis

At the end of the trial period, a histopathological examination was performed on the intestinal (duodenum) tissue samples of the experimental and control group quails. Tissue samples were fixed in 10% buffered formaldehyde and after trimming, they were transferred to tissue tracking cassettes. The cassettes were rinsed with tap water for 8–10 h and loaded into an automatic tissue tracking device (Leica TP 1020, Wetzlar, Germany). The tissues were subjected to varying degrees of alcohol, xylene, and paraffin series with an automatic program, and paraffin was blocked in a blocking device (Leica EG1150 H, Wetzlar, Germany). The paraffin blocks obtained were sectioned at 3–5 micron thickness with the help of a rotary microtome (Leica RM2125, Wetzlar, Germany) and stained with hematoxylin–eosin (H.E) method (Luna 1968) and studied under the light microscope. Microscopic evaluations were performed with an Olympus BX 43 microscope with fluorescent attachment and DP 72 camera and pictures were taken.

### 2.3 Biochemical Analysis

At the end of the trial, blood samples taken from quails in EDTA tubes during sacrification were analysed using the fully auto hematology analyser (Prokan PE‐6800, Germany).

### 2.4 Microbiological Analysis

The abdominal cavities of the quails were opened as soon as possible after they were sacrificed, and samples were taken from the intestines using sterile forceps and scissors under aseptic conditions and brought to the laboratory as soon as possible under the cold chain. Samples brought to the laboratory were opened under aseptic conditions using a sterile scalpel, and 1 g of the intestinal contents was taken. To perform microbiological analysis, decimal dilutions were prepared using 9 mL of 0.1% peptone (Merck, Darmstadt, Germany) from the intestinal content taken.

 For total mesophilic aerobic bacteria counts, plate count agar (PCA) (Merck, Darmstadt/Germany) was inoculated using the pour plate method and incubated at 35⁰C for 48 h. At the end of the incubation period, all colonies that developed on the plates were counted as total mesophilic aerobic bacteria (USDA/FSIS, 2011). Violet red bile dextrose agar (Merck, Darmstadt, Germany) was used for *Enterobacteriaceae* enumeration and inoculations were made using the pour plate method. After the first layer of the medium was added to the plates, the second layer was added. The plates were then incubated at 37±1°C for 24 h. At the end of the incubation period, typical colonies that grew 1–2 mm in diameter, red and with a ring‐shaped halo around them and that were negative for oxidase test were counted and evaluated (ISO 2004). Violet Red Bile Agar (VRB) (Merck, Darmstadt/Germany) was used to quantify coliform bacteria and the plates were incubated at 37± 1°C for 24 h. At the end of the incubation period, all dark red colonies formed on the plates were counted as coliform bacteria (ISO 2006). Tryptone bile X‐glucuronide agar (Merck, Darmstadt, Germany) was used for *Escherichia coli* enumeration. The inoculated plates were incubated at 30 ±1°C for 4 h and then at 44± 1°C for 18 h. At the end of the incubation period, the number of blue‐green coloured colonies formed on the plates was determined (ISO 2001).

### 2.5 Statistical Analysis

Firstly, assumptions of homogeneity of variance and normality were assessed using Levene's test and the Shapiro‐Wilk test, respectively. One‐way analysis of variance (ANOVA) was employed to simultaneously compare the means of biochemical and microbiological parameters across multiple groups. ANOVA is appropriate for testing the null hypothesis that all group means are equal, effectively controlling the family‐wise Type I error rate. Tukey's HSD post hoc test was applied to identify which specific groups differ. This test controls for Type I error across multiple comparisons, enabling reliable detection of significant differences between pairs of groups. Data are presented as means ± standard errors in the tables, with statistical significance set at *p* < 0.05, and all analyses were performed using SPSS 20.0 (IBM, USA).

## 3 Results

**FIGURE 1:**
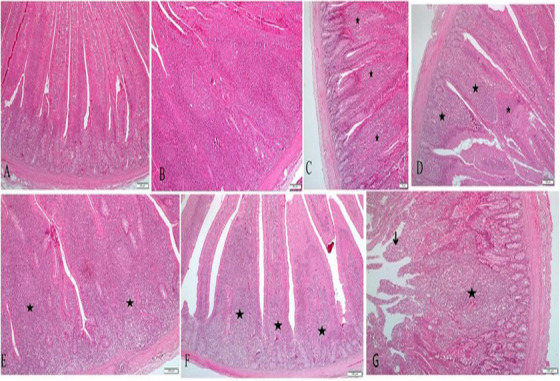
Intestinal microscopic images in control and experimental groups. (A) Control group normal histological appearance, scale bar 100 µm. (B) Diffuse severe inflammatory infiltration in the infection group, scale bar: 50 µm. (C) Inflammatory infiltration of the lamina epithelium in the infection + antibiotic group (stars), scale bar 100 µm. (D) Inflammatory infiltration in the lamina propria (stars) in the infection + feed 100 group, scale bar 100 µm. (E) Inflammatory infiltration in the lamina propria in the infection+feed 300 group (stars), scale bar 100 µm. (F) In the infection + water 100 group, diffuse inflammatory infiltration (stars) into the lamina propria and epithelium and general histological structures of the villi were preserved, scale bar 100 µm. (G) In the infection + water 300 group, lamina propria (star) and lamina epithelialis (arrow) inflammatory infiltration, scale bar 100 µm. **H.E** X 10.

As shown in Figure 1, in the examined sections, varying degrees of inflammatory reaction were detected in all groups. The most severe inflammation findings were detected in the infection group (G2) and the mildest in the control group (G1). Boric acid added to both water and feed showed antibiotic‐like effects on inflammation. The inflammatory lesions are intensely localized in the submucosa and lamina propria layers. The inflammatory response in the region was predominantly composed of lymphohistiocytic cells. Heterophil leukocytes were also found in a few sections. Although necrotic areas were observed in a few sections of intestinal villi, typical histologic structures were generally preserved in all groups, and no villous atrophy was observed (Figure 1B–G). Although inflammatory mononuclear cell infiltrations varied in severity, they were considered primary lesions in all experimental groups.

The total amount of white blood cells, lymphocytes, monocytes, and granulocytes titers and their proportional values in the blood samples taken from the study groups in the trial are presented in Table 1. Among the study groups, the highest levels of white blood cells were found in the infection group, whereas the lowest levels were found in the control group (P<0.05). Infection + Antibiotic and Infection + Boric acid groups had similar values. The groups showed similar lymphocyte, monocyte, and granulocyte titers values and proportional values in the blood (*p* > 0.05).

A significant decrease in *Enterobacteriaceae* and coliform counts in intestinal contents was observed with boric acid taken with water (G6 and G7) as compared to boric acid taken with feed (*p* < 0.01). In this regard, boric acid administered with feed did not significantly reduce the number of *Enterobacteriaceae* and coliforms compared to the infection group (*p*> 0.05). In contrast, boric acid administered with water provided a significant decrease compared to the infection group, similar to the antibiotic‐treated group (*p* < 0.01). There was a slight decrease in the number of *Enterobacteriaceae* and coliforms and in the number of *E. coli* and total mesophilic aerobic bacteria (TMAB) when boric acid was taken with water. However, these differences were insignificant compared to the infection group (*p* > 0.05). Also, comparing Group G3 (treated with antibiotics) and Group G7 (treated with boric acid in water), the effect sizes indicate negligible in bacterial populations, except for the Coliform group, which exhibits a large effect size (1.394). *Enterobacteriaceae* shows a tiny effect size (0.098), suggesting minimal variation between the two treatments. Similarly, *E. coli* and TMAB display small effect sizes (0.442 and 0.402, respectively), indicating only modest results. These findings suggest that boric acid administered via water and antibiotics exerts similar effects on *Enterobacteriaceae*, *E. coli*, and TMAB, while a more pronounced difference is observed in Coliform bacteria.

## 4 Discussion

Multidrug‐resistant bacteria pose a significant public health threat, as the rise in antimicrobial resistance, combined with the limitations of current preservatives, has intensified the search for new and effective antimicrobial agents. Given that antibiotic resistance severely complicates infection treatment, researchers are increasingly exploring alternative strategies, ranging from the natural elimination of resistant bacteria by predatory microorganisms such as *Bdellovibrio bacteriovorus* to the use of natural compounds like polyphenols, which inhibit pathogen proliferation and slow disease progression. Beyond these biological approaches, boron has recently emerged as a promising candidate due to its diverse beneficial properties, including anti‐cancer, anti‐viral, anti‐inflammatory, and therapeutic effects (Barbol et al. 2024; Celebi et al. 2024; Solfaine et al. 2024). However, excessive boron intake has been linked to cellular injury and toxicity in various animals and humans. Specifically, in Japanese quails, appropriate supplemental boron levels effectively reduced weight gain, feed consumption, and feed efficiency (Khaliq et al. 2018).

In this study, boron supplementation demonstrated a significant reduction in intestinal lesions comparable to the antibiotic‐treated group, which can be attributed to its antibacterial properties. The infection‐induced group (G2) exhibited significantly more severe and widespread inflammatory reactions than the other groups. Correspondingly, white blood cell counts were significantly higher in the infection group than in the control group (Table 2). The improvements observed in the antibiotic and boric acid groups and the supportive effect of boric acid on the immune system, as reflected in intestinal findings, were attributed to its antimicrobial activity. The anti‐inflammatory effects of boric acid are primarily attributed to its ability to inhibit the activation of the NF‐κB signalling pathway, a key regulator of inflammation. By preventing NF‐*κ*B translocation to the nucleus, boric acid reduces the expression and release of pro‐inflammatory cytokines such as TNF‐α and IL‐6. Additionally, it suppresses oxidative bursts in leukocytes and modulates the activity of overactive neutrophils, which are responsible for clearing debris and pathogens outside the vascular system. This coordinated suppression of inflammatory mediators helps to control excessive immune responses, minimize tissue damage, and preserve tissue integrity during inflammatory challenges (Pizzorno 2015; Tekeli et al. 2021; Chen et al. 2024).

**TABLE 2:** Biochemical analyses of some immune parameters of the research groups.

				Boric acid + infection				
Parameters	Control (G1)	Infection (G2)	Antibiotic + infection (G3)	Feed 100 (G4)	Feed 300 (G5)	Water 100 (G6)	Water 300 (G7)	*p* value
White blood cell, 10^3^/µL	111.08 ± 7.16^b^	134.50 ± 2.60^a^	126.30 ± 5.08^a,b^	130.45 ± 3.16^a,b^	123.60 ± 5.72^a,b^	125.40 ± 3.33^a,b^	127.98 ± 4.19^a,b^	0.046
Lymphocyte, 10^3^/µL	103.20 ± 6.31	123.60 ± 1.95	126.82 ± 9.91	127.60 ± 9.63	113.45 ± 3.49	113.05 ± 2.92	117.72 ± 3.78	0.070
Monocyte, 10^3^/µL	4.92 ± 0.58	6.82 ± 0.54	5.70 ± 1.06	7.15 ± 1.17	5.55 ± 1.16	6.07 ± 0.73	5.92 ± 0.50	0.520
Granulocyte, 10^3^/µL	2.96 ± 0.44	4.08 ± 0.81	3.77 ± 1.24	5.70 ± 2.38	4.60 ± 1.19	6.27 ± 1.56	4.34 ± 0.45	0.537
Lymphocyte, %	92.96 ± 0.57	91.86 ± 0.75	92.62 ± 1.41	90.22 ± 2.50	91.92 ± 1.48	90.15 ± 1.49	91.92 ± 0.51	0.662
Monocyte, %	4.34 ± 0.29	5.00 ± 0.28	4.40 ± 0.62	5.40 ± 0.80	4.35 ± 0.70	4.75 ± 0.45	4.52 ± 0.25	0.702
Granulocyte, %	2.70 ± 0.29	3.14 ± 0.59	2.97 ± 0.81	4.37 ± 1.72	3.72 ± 0.79	5.10 ± 1.26	3.56 ± 0.38	0.519

^a,b^The difference between the means expressed with different letters in the same row is significant. G4–G5: boric acid added to feed at 100 and 300 mg/kg, respectively; G6–G7: boric acid added to water at 100 and 300 mg/L, respectively.

In the present study, the effects of boric acid on bacterial load were examined (Table 3), revealing a significant reduction in coliform bacteria within the *Enterobacteriaceae* family in the boron groups receiving water supplementation (G6, G7), comparable to the antibiotic‐treated group (G3). Additionally, slight decreases in *E. coli* and TMAB were observed in these water‐supplemented boric acid groups. Boric acid administered via drinking water exhibited greater antibacterial efficacy than feed supplementation, likely due to its higher solubility, bioavailability, and more direct interaction with intestinal pathogens. Furthermore, its homogeneous distribution in water ensures consistent dosing among animals, minimizing individual variation in intake and therapeutic response (Vermeulen et al., 2002). Boric acid exerts its antimicrobial effect by diffusing through microbial cell membranes and disrupting both enzymatic and non‐enzymatic processes. Specifically, it has been shown to inhibit NADH oxidation, which reduces metabolic activity and ultimately results in microbial death (Hunt 1998). Consistent with these findings, other studies have demonstrated that boric acid applied through various methods effectively reduces microbial loads in poultry. Similarly, Hernandez‐Patlan et al. (2018) reported vigorous antimicrobial activity of boric acid against Salmonella enteritidis. Similarly, Eroglu et al. 2024, Eroglu et al. 2025) disinfected goose eggs using boric acid via washing and spraying, resulting in significant reductions in total mesophilic aerobic bacteria (TMAB), enterobacteriaceae, total coliforms, and *Escherichia coli* on the egg surfaces. Additionally, Cengiz et al. (2018) showed that boric acid supplementation in poultry diets reduced both litter pH and NH3 volatilization by controlling microbial activity.

**TABLE 3:** The Sekal bacterial profile in the groups.

				Boric acid + infection				
Parameters	Control (G1)	Infection (G2)	Antibiotic + infection (G3)	Feed 100 (G4)	Feed 300 (G5)	Water 100 (G6)	Water 300 (G7)	*p* value
*Enterobacteriaceae*	2.09 ± 0.09^c^	3.55 ± 0.19^a,b^	3.12 ± 0.31^b^	4.20 ± 0.25^a^	3.54 ± 0.25^a,b^	3.13 ± 0.22^b^	3.20 ± 0.12^b^	0.004
Coliform	2.10 ± 0.15^c^	3.29 ± 0.22^a^	2.36 ± 0.13^b,c^	3.53 ± 0.17^a^	3.21 ± 0.23^a^	2. 0.02^b^	2.90 ± 0.09^b^	0.009
*E. coli*	2.07 ± 0.12^b^	2.99 ± 0.31^a^	2.86 ± 0.29^a,b^	3.59 ± 0.23^a^	3.11 ± 0.23^a^	2.86 ± 0.38^a,b^	2.36 ± 0.36^a,b^	0.021
TMAB	4.58 ± 0.48^b^	6.00 ± 0.21^a^	4.94 ± 0.06^a,b^	5.47 ± 0.17^a,b^	5.20 ± 0.13^a,b^	5.05 ± 0.17^a,b^	5.28 ± 0.34^a,b^	0.013

^a,b,c^The difference between the means expressed with different letters in the same row is significant. TMAB: G4–G5: boric acid added to feed at 100 and 300 mg/kg, respectively; G6–G7: boric acid added to water at 100 and 300 mg/L, respectively.

These findings collectively demonstrate the potential of boron‐containing compounds for developing novel antibacterial and antiseptic agents that can be applied to treating bacterial infections and environmental disinfection. Therefore, boron compounds hold enormous untapped potential in medicine (Koldemir‐Gündüz et al., 2021). Besides, boric acid has been shown to lower intestinal IgA levels, leading to better gut health. Idiz et al. (2019) found that using boric acid supplemented with ampicillin was more effective than using ampicillin alone in their model with intra‐abdominal sepsis‐induced rats. Raimondi et al. (2006) revealed that different boron derivatives have bactericidal and fungicidal effects on *Staphylococcus aureus* ATCC 25923, *E. coli ATCC 25922*, and *Candida albicans* ATCC 10231 under in vitro conditions. Yılmaz (2012) found that boric acid and sodium tetraborate were bactericidal against *Staphylococcus aureus* ATCC 25923, *Acinetobacter septicus* DSM 19415, *Escherichia coli* ATCC 35218, and *Pseudomonas aeruginosa* ATCC 27853 bacteria in vitro, while Szałaj et al. 2018) found that boronic esters esterified with lipopeptides showed a good antibacterial effect against *Escherichia coli* by inhibiting *Escherichia coli* type I signal peptidase (EcLepB). Furthermore, Kıvanç et al. (2018) suggested that hexagonal boron nitrite nanoparticles had a significant antimicrobial effect against *streptococcus mutans* 3.3, *Staphylococcus pasteuri* M3, *Candida sp* M25, and *Streptococcus mutans* ATTC 25175 and prevented biofilm formation in the mouth by impeding the development of *streptococcus mutans 3.3*, *Streptococcus mutans ATTC 25175*, and *Candida sp M25*. Likewise, the reduction in the number of coliform bacteria in boric acid groups in this study may be an indication that boric acid supplementation is significantly effective against coliform bacteria, boosts the immune system due to its antimicrobial effect, and has a healing effect on the intestinal flora by suppressing coliform bacteria.

## 5 Conclusions

In conclusion, boric acid administration through both feed and water demonstrated efficacy against intestinal pathogens, with markedly greater effectiveness observed via water supplementation. As a natural, cost‐effective, and easily applicable alternative, boric acid presents a promising strategy to address the growing challenge of antibiotic resistance—offering benefits such as improved animal health, reduced treatment costs, lower antibiotic residues, and decreased environmental burden. These findings hold practical value for livestock producers and policy‐makers seeking sustainable and responsible approaches to antimicrobial use in animal production systems.

## Author Contributions

**Sultan Aslan**: conceptualization, investigation, writing–original draft, writing–review and editing, methodology, formal analysis, funding acquisition, visualization, project administration. **Ulku G. Simsek**: conceptualization, investigation, funding acquisition, writing–original draft, methodology, visualization, writing–review and editing, project administration, formal analysis. **Hatice Eroksuz**: methodology, investigation, formal analysis. **Burcu Karagulle**: methodology, investigation, formal analysis. **Mehmet EROGLU**: investigation, writing–review and editing, formal analysis, visualization. **Gokhan K. Incili**: investigation; methodology, formal analysis. **Canan A. Incili**: investigation, methodology, formal analysis.

## Ethics Statement

Ethical approval was obtained from Elazığ Veterinary Control Institute Animal Experiments Local Ethics Committee with the decision dated 14/12/2018 and numbered 2018/05.

## Conflicts of Interest

The authors declare no conflicts of interest.

## Data Availability

The data presented in this study are available on request from the corresponding author.
